# Metabolite biomarkers present in urine predict alterations in skeletal muscle associated with sarcopenia

**DOI:** 10.3389/fragi.2026.1736916

**Published:** 2026-03-20

**Authors:** Rafaela Andrade-Vieira, Hirad A. Feridooni, Alyne Teixeira, Dylan Deska-Gauthier, Xingjian Chang, Tobias Karakach, Rebecca Moyer, John P. Frampton

**Affiliations:** 1 Myomar Molecular Inc., Halifax, NS, Canada; 2 Faculty of Medicine, Department of Biochemistry & Molecular Biology, Dalhousie University, Halifax, NS, Canada; 3 Faculty of Medicine, Department Pharmacology, Dalhousie University, Halifax, NS, Canada; 4 Faculty of Health, School of Physiotherapy, Dalhousie University, Halifax, NS, Canada; 5 Faculty of Medicine, School of Biomedical Engineering, Dalhousie University, Halifax, NS, Canada

**Keywords:** aging, metabolomics, musculoskeletal health, sarcopenia, urinary biomakers, metabolomic and musculoskeletal health

## Abstract

**Introduction:**

Sarcopenia, characterized by the progressive loss of skeletal muscle mass and strength with age, is associated with adverse health outcomes and reduced health span in aging populations. Early detection is critical for implementing preventive strategies; however, current diagnostic methods are often costly, specialized, and not suitable for routine screening. This study aimed to identify metabolite biomarkers associated with early alterations in muscle metabolism that may support accessible screening approaches.

**Methods:**

In this cross-sectional observational study, serum samples from 200 individuals aged 550–70 years from the general population were analyzed. Participants completed the International Physical Activity Questionnaire (IPAQ). In a subset of 60 participants, urine samples were also collected, and participants underwent additional assessments including the Short Physical Performance Battery (SPPB), dual-energy X-ray absorptiometry (DXA), IPAQ, and a supplementary health questionnaire. Targeted metabolomic analyses were performed to identify metabolites associated with early sarcopenia-related metabolic changes.

**Results:**

A panel of metabolites in serum—L-glutamic acid, xanthine, taurine, succinate, and L‐carnitine—was associated with early alterations in muscle metabolism. These metabolites were also detectable in urine samples. Importantly, predictive performance for sarcopenia-related changes was observed when the metabolites were analyzed as a combined panel rather than as individual biomarkers.

**Conclusion:**

Our findings identify a metabolite panel detectable in urine that reflect early metabolic alterations associated with sarcopenia. This panel provide a foundation for developing accessible screening tools to support early detection and preventive strategies for muscle health decline in aging populations.

## Introduction

Muscle health plays a pivotal role in human wellbeing, influencing physical performance, mobility, longevity, and quality of life. As skeletal muscle ages, it undergoes structural and functional changes associated with physical limitations and increased risk of disease. Sedentary lifestyle, inadequate exercise, and poor nutrition increase the risk of developing sarcopenia, a progressive loss of skeletal muscle mass and strength that leads to functional decline and increased risk of adverse health outcomes (Dent et al., 2021). Studies have shown that reduced physical activity contributes to muscle atrophy, impaired metabolism, and decreased strength, while higher activity levels are linked to a lower prevalence of sarcopenia in older adults (Petermann-Rocha et al., 2022; Rogeri et al., 2021). It is estimated that sarcopenia affects 10%–16% of the aging population, but rates may be higher as sarcopenia is not easily diagnosed (Petermann-Rocha et al., 2022; Yuan and Larsson, 2023). By 2050, the proportion of the global population over 60 years of age is projected to be 22% and muscle health will be key to ensuring their quality of life and overall health.

With age-related muscle loss contributing to reduced quality of life and increased injury risk, early detection of sarcopenia and accurate monitoring of muscle health will become critical for promoting healthy aging and longevity. However, the ability to accurately assess muscle health and detect sarcopenia at early stages remains challenging. Magnetic resonance imaging (MRI) and dual-energy X-ray absorptiometry (DXA) are considered the most reliable non-invasive methods for assessing muscle mass (Beaudart et al., 2016). Unfortunately, MRI and DXA are not commonly available in primary care settings due to the need for expensive and non-portable equipment requiring highly trained personnel for operation (Yuan and Larsson, 2023). To address the limitations of traditional diagnostic methods, physical performance tests such as the Short Physical Performance Battery (SPPB) and grip strength assessment are commonly employed in primary care settings to evaluate muscle health. The SPPB is a standardized tool designed to provide an objective, reproducible assessment of lower extremity function. It has been validated across diverse populations and is widely used in clinical and research settings to evaluate frailty, disability, and mortality (Cruz-Jentoft et al., 2019; Phu et al., 2020; Bernabeu-Mora et al., 2015). Grip strength assessment, while not part of the SPPB, is considered a measure of overall muscle strength. It is widely used in sarcopenic patients due to its strong correlation with functional decline and adverse health outcomes (Cruz-Jentoft et al., 2019). Although physical assessments can assist in screening for sarcopenia, these tests can be confounded by the motivation and cognition levels of patients, and do not reliably detect pre-sarcopenia, which is characterized by a loss of muscle mass preceding the loss of muscle strength and performance (Beaudart et al., 2016; Cruz-Jentoft et al., 2019; Goates et al., 2019).

To overcome the current issues of access to and sensitivity of existing approaches for the early detection of sarcopenia and pre-sarcopenia in primary care settings, there has been an intensive effort to identify biomarkers of sarcopenia present in various biofluids, most commonly in serum (Qaisar et al., 2021; Kameda et al., 2021; Ying et al., 2022). In urine, biomarkers for the detection of sarcopenia have yet to be revealed. Creatinine in urine has been used as a proxy for muscle mass, but this biomarker alone cannot reliably predict muscle atrophy (Thongprayoon et al., 2016; Kashani et al., 2017; Patel et al., 2013; Heymsfield et al., 1983). Recent efforts have therefore focused on identifying additional metabolic markers that predict sarcopenia, for example, markers involved in energy-related signaling associated with muscle fiber degradation and atrophy in sarcopenia (Pes et al., 2001; Vasconsuelo et al., 2013) and markers associated with mitochondrial dysregulation (Chabi et al., 2008; Iqbal and Hood, 2014; Trounce et al., 1989). Monitoring intrinsic changes to muscle metabolism could provide valuable insights not only into muscle mass but also health (overall muscle mass and function), thereby facilitating, accurate diagnosis of sarcopenia, better triaging of patients and disease prevention. Nonetheless, a comprehensive understanding of the relationship between muscle metabolism and sarcopenia remains elusive.

The objectives of this study were to identify a unique metabolite panel in both serum and urine capable of detecting sarcopenia. Our findings were cross validated with the gold standard methods of diagnosing sarcopenia (DXA and physical assessment). By adopting this approach, we demonstrate the potential for a panel of metabolites present in urine to detect changes in muscle metabolism, providing more comprehensive analysis and monitoring of muscle health.

## Methods

### Study design and population

Biological samples were collected by the Atlantic Path Tissue Bank and was shared in a non-identifiable format. Evaluation of serum and urine samples from the tissue bank was performed with ethics review exemption by the Nova Scotia Health Authority Research Ethics Board (REB file #: 1027579). The bank provided 200 serum samples and 80 urine samples along with corresponding scores from the International Physical Activity Questionnaire (IPAQ) long-form, age (50-70 years old, average age of 61) and sex data. All urine samples were frozen and stored at −80 °C by the Atlantic Path Tissue Bank and analyzed together on a later date.

In the prospective clinical evaluation study conducted at Newfoundland Health (St. John’s, NL, Canada; n = 60), fresh urine samples were collected from 30 males and 30 females aged 50-70 years on the same day they underwent physical performance tests using the SPPB and grip strength. DXA scans were scheduled around the same time to ensure that measurements of muscle mass closely corresponded with the urine sample collection and physical assessment. For the Newfoundland cohort, all urine samples were collected in the morning after an overnight fast (≥8 h), using the participants’ first-morning void. Samples were immediately frozen at −80 °C. To minimize batch variability, all samples were thawed simultaneously and analyzed together on a later date. Participants were allowed to have breakfast before completing the physical assessments. This study also included IPAQ and a demographics questionnaire. The study process is summarized in Supplementary Figure S1. Sample sizes were determined based on the availability of biospecimens from the Atlantic Path Tissue Bank and feasibility of recruitment within the study period for the Newfoundland cohort. This sample size is consistent with prior exploratory biomarker studies designed to identify metabolic patterns.

The exclusion criteria for the Tissue Bank samples and the Newfoundland cohort were kidney disease, diabetes (type I and type II), muscular dystrophies, Parkinson’s disease, multiple sclerosis, and amyotrophic lateral sclerosis. Individuals with kidney disease were excluded due to its potential to impair renal function. Additionally, individuals with muscular dystrophies, amyotrophic lateral sclerosis and neurological disorders Parkinson’s disease, multiple sclerosis were excluded, as these conditions can alter muscle metabolism, degradation, and function, potentially influencing metabolite levels in urine. For descriptive purposes, musculoskeletal disease was defined as self-reported arthritis, osteoarthritis, or osteoporosis.

Participants in the Newfoundland Study who were unable to complete the DXA scan or perform the sit-stand test were excluded. Recruitment and data collection was performed between November 2022 and May 2023. Participants provided written consent. The study was reviewed and approved by the ethics committee of Newfoundland Health Services (REB#20230736). To minimize bias, all samples were collected under standardized conditions, processed in single analytical batches, and participants with confounding comorbidities were excluded.

### International Physical Activity Questionnaire

The IPAQ long-form was used to assess the physical activity levels of participants. Briefly, the IPAQ is a validated tool designed to measure physical activity across four domains (work related activity, transportation, domestic and gardening activities, and leisure-time physical activity, as described previously (Booth, 2000). Participants reported the frequency (days per week) and duration (minutes per day) of physical activities at different intensity levels (walking, moderate, and vigorous). Categorical summaries were used to classify participants into three physical activity levels following the IPAQ scoring protocol (low activity, moderate activity, and high activity). Individuals classified as low activity by IPAQ were assigned to the sedentary group in our study, while individuals classified as high activity by IPAQ were assigned to the active group.

### Metabolomics

Serum samples were analyzed using liquid chromatography-mass spectrometry (LC/MS) on a QTRAP 5500 instrument (SCIEX). We used a targeted method allowing for the measurement of 500 metabolites. For sample preparation, 20 µL serum samples were combined with 85uL of 99.8% methanol (containing isotope-labeled internal standards). Samples were then centrifuged at 10,000 RCF for 5 min and the supernatant was collected for analysis. The samples were not dried or lyophilized before LC/MS analysis. To ensure consistency and minimize potential batch effects, all serum samples were processed in one batch sequentially without interruption on the LC/MS system. Internal controls were placed every 20 samples to monitor sample stability and verify data integrity. Quality was assured by pooling 20 uL from each sample for inclusion in the analysis, with peaks measured at fifty-point intervals. Data were collected and analyzed using Compound Discoverer (Thermo Scientific), and statistical analysis and graph construction were completed in MetaboAnalyst (version 6, McGill University, Montreal, QC, Canada) and Python (3.11.8). Metabolite peaks were identified by analyzing each biomarker and removing background noise using Compound Discoverer.

Principal component analysis (PCA) was applied to LC/MS peak intensities from serum samples to identify difference between active and sedentary individuals using MetaboAnalyst. Significant differences in serum metabolite levels between active and sedentary individuals were assessed using Wilcoxon Mann-Whitney t-tests. Pathway enrichment analysis was conducted using MetaboAnalyst software to identify metabolic pathways associated with muscle health. The analysis was performed on biomarkers that exhibited significant differences between active and sedentary groups. The enrichment tool in MetaboAnalyst utilized the Kyoto Encyclopedia of Genes and Genomes (KEGG) database to map metabolites to their corresponding biochemical pathways. The enrichment ratio was calculated based on the observed frequency of significant biomarkers in each pathway relative to the expected frequency in the full metabolic background. To visualize differences in serum metabolites between sedentary and active groups for males and females, volcano plots were generated. Each plot displays the log_2_ fold change on the x-axis and the–log_10_ (p-value) on the y-axis, with sedentary individuals used as the reference group. This approach highlights metabolites with both strong magnitude and statistical significance, aiding in the selection of biomarkers for downstream pathway analysis.

Metabolite expression heatmaps were generated separately for male and female participants using Python within a Jupyter environment. For all statistically significant metabolite, z-score normalization was applied across all samples to standardize values (mean = 0, standard deviation = 1), using the Standard Scaler function from Scikit-learn v1.4.2. Heatmaps were constructed using Seaborn v0.11.2 and Matplotlib v3.6.3, employing a diverging RdBu_r color palette centered at zero, with a scale range from −2 (downregulated) to +2 (upregulated).

### Metabolic assays

Urine samples from the Atlantic Path Tissue Bank and Newfoundland Health study were analyzed with the following metabolic assays: xanthine (Abcam cat # ab155900), taurine (Abcam cat # ab241040), succinate (Abcam cat # ab204718), L-glutamic acid (Elabscience cat # E-BC-K903-M) and L-carnitine (Abcam cat # ab83392), following the manufacturer’s instructions. Briefly, all urine samples were centrifuged at 10,000 RCF for 10 min, and the supernatant was collected. For each assay, a sample volume of 50 µL of the urine supernatant was added to a designated 96-well microplate, followed by the addition of 50 µL of reaction mix (comprising the assay buffer, enzyme mix, developer, and/or probe) or background control (comprising the assay buffer, developer, and/or probe). The microplate was then incubated at room temperature for 30 min. Absorbance was measured for xanthine (595 nm), taurine (414 nm), succinate (450 nm), and L-glutamic acid (450 nm), and fluorescence was measured for L-carnitine (535/587 nm excitation/emission) according to the manufacturer’s instructions for each assay using a FilterMax F5 Multi-Mode Microplate Reader (Molecular Devices, California, United States of America).

Following two-way ANOVA, Tukey’s multiple comparisons test with a single pooled variance was used for *post hoc* pairwise comparisons to analyze differences in metabolite concentrations. A p-value of less than 0.05 was considered statistically significant. Multiple logistic regression analysis was performed to generate receiver operating characteristic (ROC) curves and analyze the area under the curve (AUC). ROC curves were used to evaluate the predictive accuracy of the metabolite biomarker panel for physical activity and/or sarcopenia. ROC curves plot sensitivity (true positive rate) against 1 – specificity (false positive rate). The AUC quantifies the overall diagnostic performance, with values closer to 1 indicating excellent discrimination (Park et al., 2004). Urine dilution was assessed using urine density measurements obtained with a handheld digital density meter (DMA 35 Basic, Anton Paar GmbH, Graz, Austria). The instrument measures density based on the oscillating U-tube principle with a resolution of 0.001 g/cm^3^ and repeatability of 0.0005 g/cm^3^. Urine density showed minimal inter-individual variability across participants (mean 1.01 ± 0.004 g/cm^3^), indicating comparable hydration status and no evidence of systematic dilution differences. Given the narrow distribution of density values and the exclusion criteria, metabolite concentrations were not further normalized to creatinine or dilution metrics in this cohort. Furthermore, creatinine normalization was not applied, as urinary creatinine may be influenced by age, sex, and skeletal muscle mass, which may introduce bias in sarcopenia-focused cohorts (Baxmann et al., 2008).

### Short Physical Performance Battery and grip strength

The SPPB was used to assess the physical performance of the participants including a gait speed test, a 5-time sit and stand test, and a timed stance test. In addition to the SPPB, a grip strength test was included as a measure of physical performance.

*Gait speed test*: participants were instructed to walk at their usual pace along a straight 4-m walkway. A stopwatch was used to time how long it took for the participants to complete the walk. The time recorded for their second gait speed test was used for analysis and data were reported in meters per second.

*Sit and stand test*: participants were asked to sit on a chair with their feet flat on the ground. Then, they were instructed to stand up and return to a seated position five times as quickly as they could with arms crossed over their chests. The time to complete the test was recorded for analysis.

*Timed Up & Go (TUG)*: participants were asked to stand up from a chair and walk 3 m, then return to the chair and sit. The time required to complete the task was recorded.

*Grip strength test:* Grip strength was measured using a dynamometer (Baseline STD hydraulic hand dynamometer 12-0240). Measurements were recorded in kg. Grip strength was recorded twice for each hand, and if there was a large discrepancy between the two readings (greater than a kg), a third set of measurements was taken. Measurements from both dominant and non-dominant hands were averaged for analysis.

### Dual-energy X-ray absorptiometry

DXA was used to assess appendicular lean mass index (ALMI) as a measure of muscle mass. Appendicular lean mass (ALM) was calculated as the sum of the lean soft tissue mass from all four limbs and was expressed in kilograms. ALM values were not averaged or expressed as a percentage. ALMI was then derived by dividing ALM by height squared (kg/m^2^), following European Working Group on Sarcopenia in Older People (EWGSOP) guidelines (Cruz-Jentoft et al., 2019). The equipment used was a General Electric (GE)/Lunar scanner Prodigy Advance with software version 13.6. Patients were properly positioned in the scanner and a total body scan was performed according to the GE standardized protocol.

### Sarcopenia status

Sarcopenic status was assigned to participants who met at least one of the following criteria: 1) SPPB score <6, consistent with EWGSOP2 guidance indicating severe impairment in physical performance (Cruz-Jentoft et al., 2019), 2) ALMI values of <5.67 kg/m^2^ for females and <7.26 kg/m^2^ for males (Cruz-Jentoft et al., 2019), and 3) grip strength cutoffs for sarcopenia was <16 kg for females and <27 kg for males according to EWGSOP2 (Cruz-Jentoft et al., 2019).

### Statistical analysis

Unless otherwise noted above, data analysis and visualization were performed using R v4.3.1 (Beagle Scouts) and Prism 10 (GraphPad Version 10.1.1 270). Multiple logistic regression was performed in Prism 10 to model the probability of the outcome as a function of urinary metabolite concentrations. The log-odds of the outcome were modeled using the following equation:

$$\mathrm{Log}\;\left(\frac{p}{1-p}\right)=\beta_{0}+\beta_{1}Taurine+\beta_{2}Xanthine+\beta_{3}L-Carnitine+\beta_{4}Succinic\;Acid+\beta_{5}Glutamic\;Acid$$

where *p* represents the probability of the outcome of interest. Model coefficients were estimated using maximum likelihood estimation. To assess model robustness in the context of limited events, leave-one-out cross-validation (LOO-CV) was performed in GraphPad Prism by iteratively refitting the multivariable logistic regression model with one participant excluded per iteration and aggregating held-out predicted probabilities to generate a cross-validated ROC curve and AUC. The estimated coefficients were: intercept = −3.330, Taurine = 0.002866, Xanthine = 0.02789, L-Carnitine = 1.365, Succinic Acid = 0.01630, and Glutamic Acid = −0.001183.

## Results

### Metabolomic analysis reveals metabolite variations between active and sedentary individuals

**FIGURE 1:**
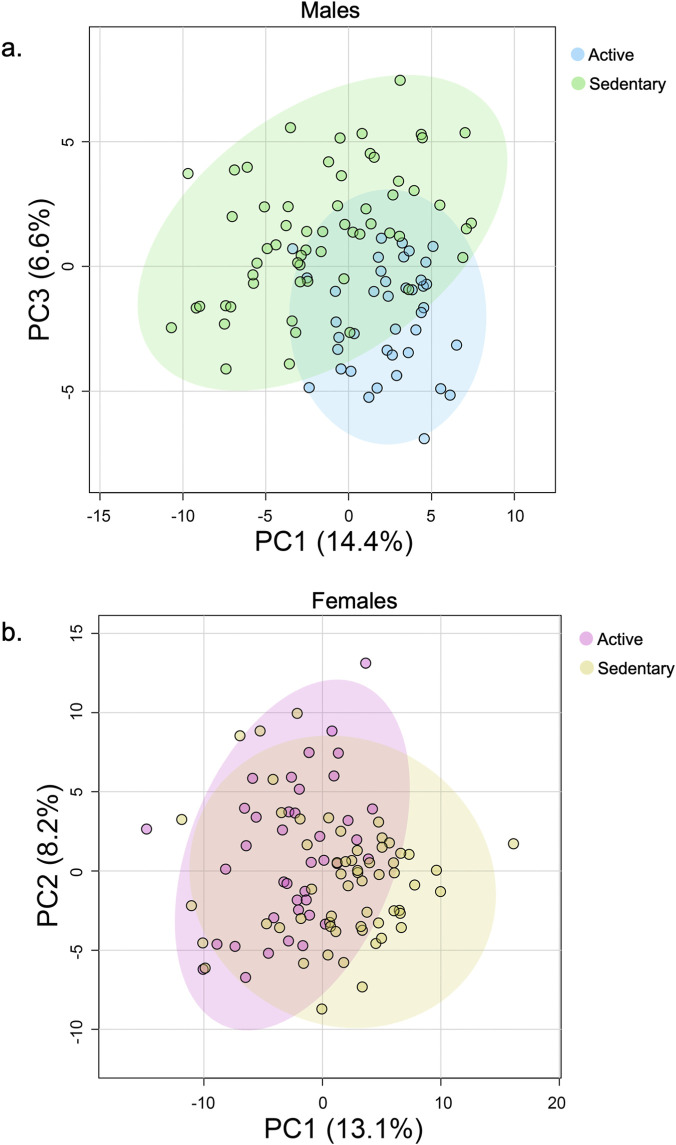
PCA score plots display distinct clustering patterns between active and sedentary groups for both males and females. **(a)** Serum samples from male participants were analyzed using LC/MS and categorized by physical activity levels based on IPAQ scores. Male participants were divided into active and sedentary groups. **(b)** Serum samples from female participants were analyzed using LC/MS and categorized by physical activity levels based on IPAQ scores. Female participants were divided into active and sedentary groups.

To explore the impact of physical activity on metabolite profiles, equal numbers of serum samples were obtained from the Atlantic Path tissue bank and categorized into active and sedentary groups based on IPAQ scores. Analyses were conducted separately for males and females. Males (n = 100) were classified into active and sedentary groups and females (n = 100) were classified similarly. PCA of metabolite peak intensities was performed to identify differences in metabolic profiles between active and sedentary individuals. The resulting score plots showed separation between active and sedentary groups for both males (Figure 1a) and females (Figure 1b). In males, principal component 1 (PC1) and principal component 3 (PC3) explained 14.4% and 6.6% of the variance, respectively. In females, PC1 and PC2 accounted for 13.1% and 8.2% of the variance, respectively (Figure 1b). These components were selected as they provided the best separation between individuals classified as active and sedentary based on IPAQ scores. The principal components represent combinations of metabolites that contribute most to the variance observed in the dataset. Specifically, PC1 captures the largest variance, potentially reflecting differences in energy metabolism, while PC2 and PC3 may represent variations in amino acid metabolism and oxidative stress pathways. Although the total variance explained by these components is modest (21.0% in males and 21.3% in females), such levels are typical in metabolomics studies due to the high dimensionality and complexity of the data (Macias et al., 2023; Dong et al., 2022).

**FIGURE 2:**
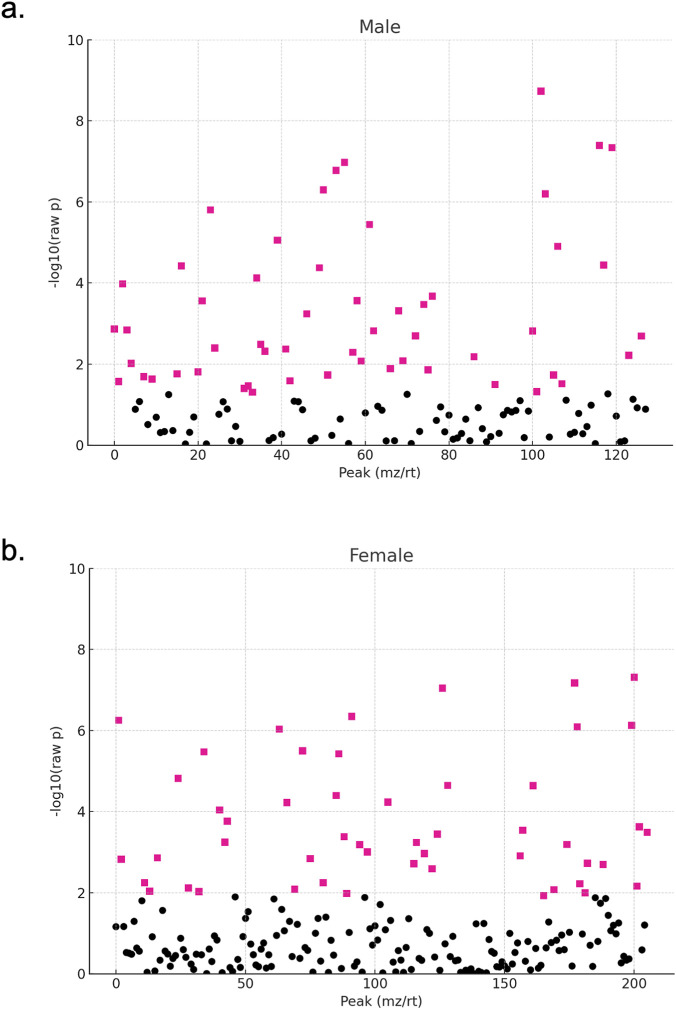
T-test analysis plots of metabolite differences between active and sedentary groups. Each point represents a unique LC/MS metabolite peak defined by its mass-to-charge ratio (m/z) and retention time (rt). The x-axis shows the index of detected peaks (mz/rt combination), and the y-axis displays the–log_10_ (raw p-value) from t-tests comparing metabolite abundance between activity groups. Pink squares indicate metabolites with significant differences (p < 0.05), while black circles represent non-significant metabolites. Results are shown separately for **(a)** males and **(b)** females, highlighting metabolic differences associated with physical activity status.

**FIGURE 3:**
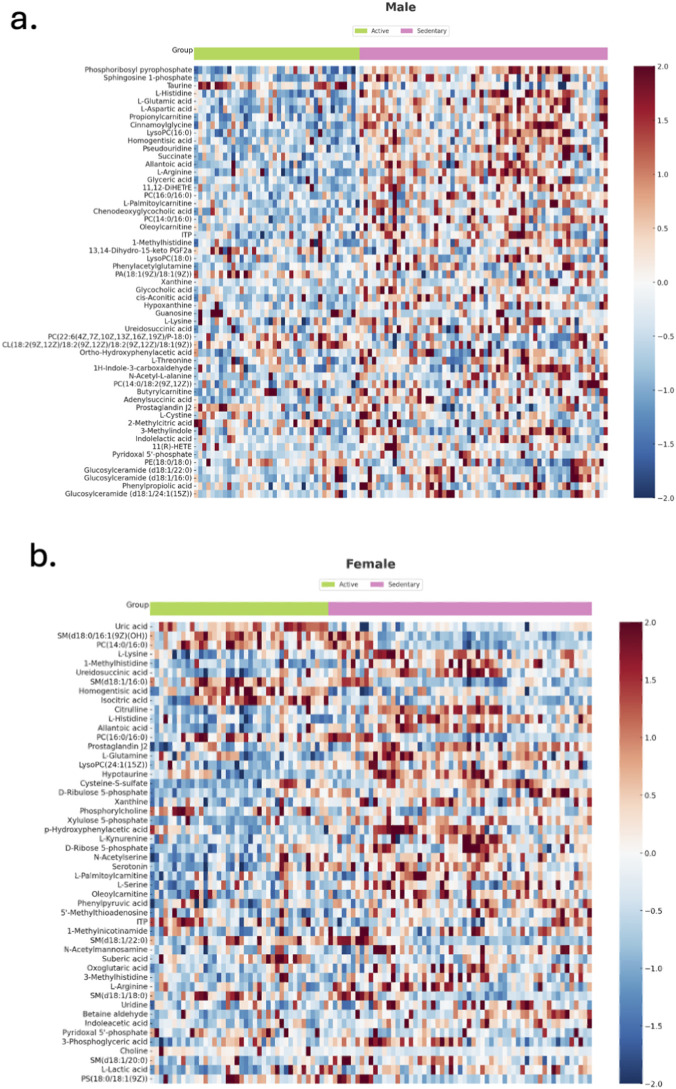
Heatmaps showing relative expression patterns generated from serum LC/MS of significant metabolites in **(a)** males and **(b)** females, comparing Active (green) and Sedentary (pink) groups. Each row represents a metabolite, and each column a participant. Metabolite values were z-score normalized across all samples. Red indicates upregulation (z > 0), and blue indicates downregulation (z < 0). Only statistically significant metabolites (based on prior hypothesis testing) are shown.

To further explore differences between active and sedentary groups, t-tests were performed for each metabolite, and the results were visualized using t-test plots. A total of 54 metabolites in males (Figure 2a) and 50 in females (Figure 2b) differed significantly between active and sedentary groups, highlighting key differences in energy metabolism. Using only the significant metabolites, we conducted heat map analysis to observe expression patterns for males (Figure 3a) and females (Figure 3b). The heat maps illustrated distinct metabolite profiles between activity levels. Metabolite abundance is shown on a color scale, where red indicates higher abundance and blue indicated lower abundance, comparing active group to the sedentary group. Abundance was measured as LC/MS peak area in arbitrary units, reflecting relative metabolite concentrations. This trend shows that sedentary individuals generally have a higher expression of most of the metabolites, especially in males. Key metabolites higher in sedentary individuals are xanthine, L-glutamic acid, L-aspartic acid and succinic acid, suggesting inefficient energy metabolism.

**FIGURE 4:**
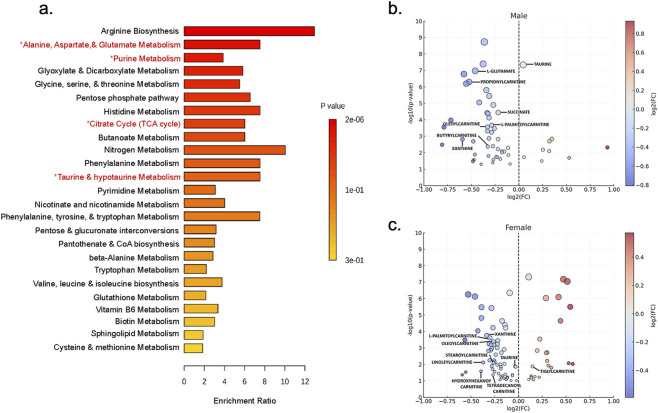
Pathway enrichment and volcano plot analyses of serum metabolites. **(a)** Pathway enrichment analysis of metabolites significantly altered between active and sedentary individuals. Pathways are ranked by enrichment ratio, with color intensity corresponding to statistical significance (p-value). Asterisks (*) indicate pathways containing one or more of the five selected biomarkers for urinalysis. **(b)** Volcano plot of male serum metabolites. The x-axis displays log_2_ fold change (FC), and the y-axis shows–log_10_ (p-value) comparing active and sedentary groups. Metabolites of interest are labeled, including L-glutamate, taurine, succinate, xanthine, and various carnitines. **(c)** Volcano plot of female serum metabolite data, annotated similarly to panel **(b)**. Point size corresponds to the effect size of each metabolite, with larger points indicating a greater magnitude of change between groups.

Pathway enrichment analysis identified arginine biosynthesis, alanine-aspartate-glutamate metabolism, and purine metabolism as the pathways with the highest enrichment ratios. Additionally, TCA cycle (citrate cycle), taurine and hypotaurine metabolism, and the pentose phosphate pathway were among the significantly enriched pathways (Figure 4a). Additionally, volcano plot analysis was used to visualize the significance of metabolites with respect to the magnitude of their change between active and sedentary individuals (Figures 4b,c). Each point represents a single metabolite, with log_2_ fold change (x-axis) and–log_10_ (p-value) (y-axis) from t-tests. In males, L-glutamic acid, succinate, and taurine showed significant fold change in active individuals, while in females, xanthine and several carnitines derivatives showed significant fold change in the sedentary group. Color intensity indicates direction and magnitude of change, and point size reflects effect size. Based on the enrichment analysis, fold change and significance levels, we selected biomarkers important in the mitochondria TCA cycle (succinate and L-carnitine), protein synthesis (L-glutamic acid and taurine) and purine metabolism (xanthine). Although, L-glutamic acid and succinate were not found in our volcano analysis in females, they were selected for further investigation since they are important intermediates of TCA cycle and protein synthesis, both important for muscle function. Other metabolites were also excluded based on limited detectability or poor urinalysis compatibility. Therefore, five metabolites (L-glutamic acid, taurine, xanthine, succinate and L-carnitine) were selected for targeted urinalysis and further evaluate their relationship with muscle health and activity levels.

### Metabolites present in urine as a function of physical activity level and clinical evaluation

**FIGURE 5:**
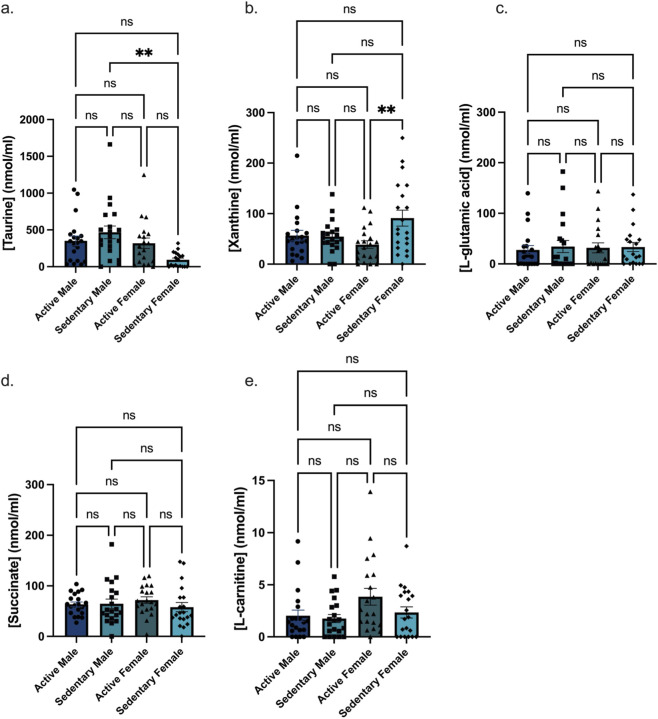
Biomarker concentrations in urine from active and sedentary male and female participants from the Atlantic Path tissue bank study. Bar graphs show the concentrations of five **(a)** taurine, **(b)** xanthine, **(c)** L-glutamic acid, **(d)** succinate, and **(e)** carnitine (nmol/mL) across four groups (active males (N = 20), sedentary males (N = 20), active females (N = 20), and sedentary females (N = 20)). Each bar represents the mean concentration ±standard error of the mean (SEM). Taurine levels were significantly higher in sedentary males compared to sedentary females (p < 0.005). Xanthine levels were significantly higher in sedentary females compared to active females (p < 0.005). No statistically significant differences were observed for L-glutamic acid, succinate, or carnitine across the groups.

**FIGURE 6:**
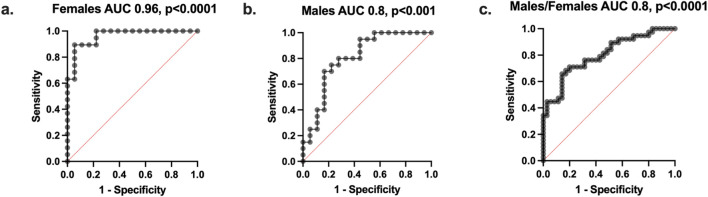
Performance of the biomarker panel for predicting levels of physical activity using IPAQ. Participants (N = 80) were first classified into two groups (active and sedentary) based on IPAQ scores. The cohort included 40 females (20 active, 20 sedentary) and 40 males (20 active, 20 sedentary). ROC curves from which area under the curve (AUC) values were calculated. **(a)** The AUC was 0.96 (P < 0.0001) with a 95% CI of 0.9–1.0 for females. **(b)** The AUC was 0.8 (P < 0.001) with a 95% CI of 0.66–0.95 for males. **(c)** The AUC was 0.8 (P < 0.0001) with a 95% CI of 0.70–0.90 for males and females combined.

To assess whether metabolic differences observed in serum between active and sedentary groups were also reflected in urine, urinalysis was performed on samples collected from the same active and sedentary individuals analyzed in the serum metabolomics cohort. Taurine levels were significantly higher in sedentary males compared to sedentary females (p < 0.005), and xanthine levels were significantly higher in sedentary females compared to active females (p < 0.005) (Figure 5). No statistically significant differences were observed for L-glutamic acid, succinate, or L-carnitine among the groups. To evaluate the combined discriminative power of the five urinary biomarkers for physical activity status, we performed ROC analysis using IPAQ-defined activity levels. In females (Figure 6a) the panel achieved an AUC of 0.96 (p < 0.0001). In males (AUC = 0.8, p < 0.001, Figure 6b), and similar predictive power when combining both sexes (AUC = 0.8, p < 0.0001, Figure 6c). These results suggest that a non-invasive urine-based assay incorporating this panel may provide a useful tool for stratifying individuals by activity level.

To further understand the relationship between these metabolites and sarcopenia, a separate cohort of individuals (N = 30 males, N = 30 females) aged 50–70 years was recruited. Participants underwent physical assessments and DXA scanning, to determine their sarcopenia status. Urine samples were collected and analyzed to evaluate the performance of the biomarker panel in differentiating sarcopenic from non-sarcopenic individuals. Table 1 summarizes participant demographics and behavioral characteristics, including physical activity levels, BMI classification, smoking status, and the presence of musculoskeletal or central neurological conditions, and cardiovascular disease. Among participants, 70% were overweight or obese, with musculoskeletal disease present in 48.3% and cardiovascular disease in 40%. Based on IPAQ assessments, 60% of the participants were categorized as active, 36.7% as moderately active, and 3.3% as inactive. Table 1 also presents key physical and DXA-based measures across sexes. Average grip strength was significantly higher (p < 0.001) in males (37.88 ± 1.85) than in females (22.55 ± 1.34). Gait speed remained consistent across sexes, suggesting no differences in measured walking performance. The average DXA ALMI was 7.69 ± 0.23 kg/m^2^ in males and 6.74 ± 0.21 kg/m^2^ in females (P < 0.05).

**TABLE 1:** Behavior and physical patterns of study participants.

​	All participants	Males	Females
# of Participants	60	30	30
Average Age (years)	59.5	59.2	59.8
IPAQ			
Inactive	5	5	0
Moderate	20	7	13
Active	35	18	17
Drink per Week			
0	13 (21.7%)	4	9
<1	15 (25%)	7	8
1-2	7 (11.7%)	4	3
3-4	11 (18.3%)	5	6
5+	14 (23.3%)	10	4
BMI categorization			
Healthy	18 (30%)	9	9
Overweight	17 (28.3%)	9	8
Obese	25 (41.7%)	12	13
Smoking			
Yes	8 (13.3%)	3	5
No	52 (86.7%)	27	25
Cardiovascular disease			
Yes	24 (40%)	12	12
No	36 (60%)	18	18
Musculoskeletal disease			
Yes	29 (48.3%)	14	15
No	31 (51.7%)	16	15
DXA & Physical Assessment			
ALMI (kg/m2)	—	7.69 ± 0.23	6.74 ± 0.21^a^
Grip strength	—	37.88 ± 1.85	22.55 ± 1.34^a^
Gait speed (m/s)	—	1.24 ± 0.03	1.31 ± 0.04
Repeated chair stand (s)	—	12.92 ± 0.42	12.41 ± 0.36
Timed up & Go (s)	—	9.07 ± 0.25	9.81 ± 0.83

^a^ Significantly different from male participants [P < 0.05, t-test: two sample assuming equal variances] for mean values = /- standard error of the mean.

**FIGURE 7:**
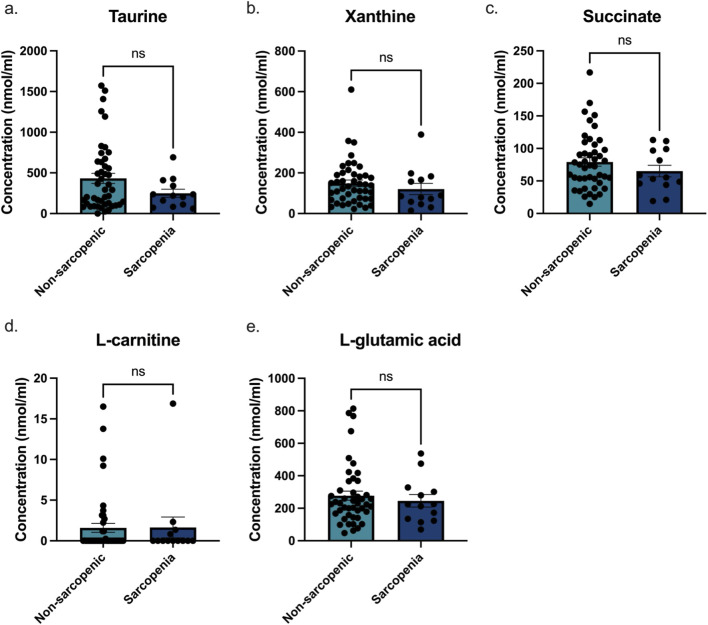
Biomarker concentrations in urine from active and sedentary male and female participants from the Newfoundland Health study. Bar graphs show the concentrations of **(a)** taurine, **(b)** xanthine, **(c)** L-glutamic acid, **(d)** succinate, and **(e)** carnitine (nmol/mL) in non-sarcopenic and sarcopenic individuals. No significant differences were observed for any of the five metabolites measured (taurine (p = 0.31), xanthine (p = 0.31), succinate (p = 0.39), L-glutamic acid (p = 0.78), and L-carnitine (p = 0.92)). Data are presented as mean ± SEM. “ns” denotes non-significance.

**FIGURE 8:**
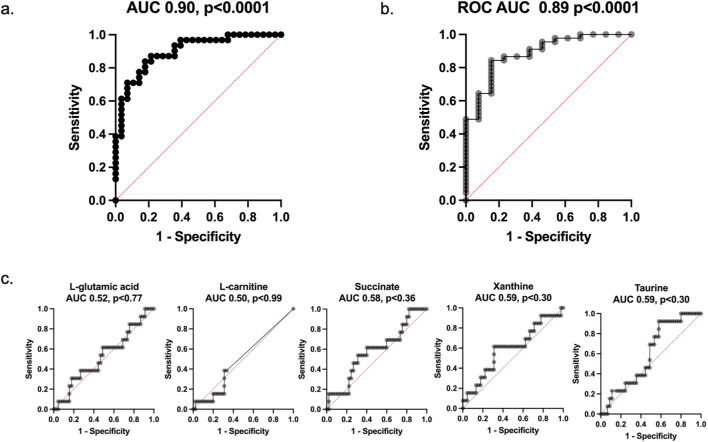
Performance of the biomarker panel and individual biomarkers for predicting sarcopenia. Ground truth (sarcopenic or non-sarcopenic status) was based on **(a)** physical assessments (AUC = 0.90, P < 0.0001) 95% CI 0.82 to 0.97, **(b)** DXA (AUC = 0.89, P < 0.0001) 95% CI of 0.82–0.99, **(c)** ROC curves for individual biomarkers, including taurine, xanthine, succinate, L-carnitine, and L-glutamic acid. Metabolites individually showed no significant predictive ability for determining sarcopenic and non-sarcopenic status.

Concentrations of taurine, xanthine, succinate, L-carnitine, and L-glutamic acid in urine were measured in non-sarcopenic (n = 45) and sarcopenic (n = 13) individuals to assess their potential as biomarkers for sarcopenia detection (Figure 7). There were no significant differences between non-sarcopenic and sarcopenic individuals for any of the individual metabolites measured. Given the absence of univariate discrimination, the metabolites were next evaluated jointly using a multivariable model. ROC curve analysis was performed to evaluate the ability of a panel of the metabolites to classify males and females based on sarcopenia status (Figure 8). The biomarker panel had a ROC AUC of 0.90 when physical assessment alone was used to determine sarcopenic status (Figure 8a). When sarcopenic status was based on DXA measurements alone, the panel had a ROC AUC of 0.89 (Figure 8b). Across LOO-CV iterations, the cross-validated AUC remained stable (mean AUC = 0.89; range 0.88–0.91) indicating that model discrimination was not driven by any single observation (Supplementary Table S1). While DXA data is a quantitative analysis of muscle mass, physical assessment is an analysis of muscle function. This data strengthens the use of our panel as they are closely related to both muscle mass and muscle function. In addition, a ROC AUC analysis of individual biomarkers (Figure 8c) revealed that xanthine (AUC = 0.59, P < 0.30), taurine (AUC = 0.59, P < 0.30), succinate (AUC = 0.58, P < 0.36), L-carnitine (AUC = 0.50, P < 0.99), and L-glutamic acid (AUC = 0.52, P < 0.77) did not show significant predictive power. In all cases individual metabolites were unreliable predictors of sarcopenic status. To confirm the ideal combination of biomarkers in our panel, we evaluated the predictive performance of every possible biomarker combination against DXA-defined sarcopenia status. The full five-marker panel achieved the highest ROC AUC of 0.89 (p < 0.0001), outperforming all partial combinations (Table 2). These results support the use of the complete panel to determine sarcopenia, as it provides the strongest classification performance relative to the DXA gold standard.

**TABLE 2:** ROC AUC values and p-values for all combinations of five urinary biomarkers in classifying sarcopenia based on DXA-defined status.

Biomarker combination	ROC AUC	P value
Taurine + L-Glutamic Acid + L-Carnitine + Succinate + Xanthine	0.89	<0.0001
Taurine + L-Glutamic Acid + L-Carnitine + Succinate	0.78	0.002
Taurine + L-Carnitine + Succinate + Xanthine	0.76	0.005
Taurine + L-Glutamic Acid + Succinate + Xanthine	0.76	0.005
L-Glutamic Acid + L-Carnitine + Succinate + Xanthine	0.71	0.020
L-Carnitine + Succinate	0.71	0.025
Taurine + L-Glutamic Acid + L-Carnitine + Xanthine	0.69	0.038
Taurine + Xanthine + Succinate	0.68	0.045
Taurine + Xanthine	0.68	0.049
Taurine + L-Carnitine + Succinate	0.67	0.058
Taurine + Succinate + L-Glutamic Acid	0.66	0.085
Taurine + Succinate	0.66	0.085
L-Glutamic Acid + Succinate	0.65	0.107
Taurine + Xanthine + L-Carnitine	0.65	0.095
Succinate + L-Glutamic Acid + L-Carnitine	0.62	0.195
Taurine + L-Glutamic Acid	0.62	0.189
Taurine + Xanthine + L-Glutamic Acid	0.62	0.176
L-Carnitine + L-Glutamic Acid + Xanthine	0.62	0.195
Xanthine + L-Carnitine	0.62	0.189
Taurine + L-Carnitine + L-Glutamic Acid	0.61	0.251
Taurine + L-Carnitine	0.61	0.236
Xanthine + L-Glutamic Acid + Succinate	0.61	0.222
Xanthine + L-Glutamic Acid	0.61	0.244
Xanthine + Succinate	0.59	0.310
Taurine	0.59	0.301
Xanthine	0.59	0.301
Xanthine + L-Carnitine + Succinate	0.58	0.376
Succinate	0.58	0.376
L-Glutamic Acid	0.53	0.773
L-Carnitine + L-Glutamic Acid	0.52	0.816
L-Carnitine	0.51	0.941

## Discussion

The objective of this study was to identify and validate metabolite biomarkers of muscle health across two independent cohorts. In the first cohort, we compared serum and urine metabolomic profiles between active and sedentary individuals to identify candidate metabolites associated with muscle metabolism. Although physical activity and sarcopenia are distinct phenotypes, they are biologically linked through shared mechanisms governing muscle mass, muscle function, and muscle metabolic activity (Hämäläinen et al., 2024; Sánchez-Sánchez et al., 2024). In the discovery phase, physical activity was used as a pragmatic proxy to identify metabolites reflecting muscle metabolic engagement and remodeling, processes that precede and contribute to sarcopenic decline. In the second, we prospectively evaluated whether these metabolites could predict sarcopenia, defined using DXA and physical performance measures. Collectively, our findings show that while individual metabolites lacked discriminatory power, the five-metabolite urinary panel comprising xanthine, taurine, succinate, L-carnitine, and L-glutamic acid demonstrated coordinated metabolic differences associated with sarcopenia when evaluated against DXA (AUC = 0.89) and physical performance measures (AUC = 0.90). These findings support the potential of using a non-invasive biomarker panel for diagnosing sarcopenia. In the next decade sarcopenia will afflict a large portion of our aging population, in addition, sarcopenia shares metabolic alterations with other muscle-related diseases, emphasizing the importance for a systemic approach to understanding and addressing muscle decline (Vasconsuelo et al., 2013; Vila et al., 2017; Janssen et al., 2016).

A key feature of sarcopenia is the preferential loss of type II (fast-twitch) muscle fibers, leading to reduced muscle strength and impaired function (Walston, 2012). This process is accompanied by fat infiltration, mitochondrial dysfunction, and increased apoptosis of myocytes all of which contribute to progressive muscle degeneration. Age-related mitochondrial decline and muscle injury further exacerbates these effects, impairing energy production, increasing oxidative stress, and reducing regenerative capacity, ultimately accelerating muscle weakening and broader health deterioration (Dent et al., 2021; Petermann-Rocha et al., 2022; Rogeri et al., 2021; Heymsfield et al., 1983; Walston, 2012; da Costa Teixeira et al., 2023).

Given the central role of muscle metabolism in these degenerative processes, it is essential to understand the molecular pathways that regulate muscle maintenance. The mTOR/AKT pathway has been linked to muscle regeneration by promoting protein synthesis and mitochondrial function, making it a key therapeutic target to reduce muscle atrophy (Pes et al., 2001; Vasconsuelo et al., 2013). Conversely, FoxO3 activation promotes the expression of atrophy-related genes, contributing to muscle degeneration when metabolic disturbances disrupt the mTOR/AKT balance (Chabi et al., 2008; Iqbal and Hood, 2014; Trounce et al., 1989). L-glutamic acid, xanthine, taurine, succinate, and L-carnitine all directly or indirectly with the mTOR pathway (Centeno et al., 2024; Jewell et al., 2013; Wu et al., 2021).

We found that these biomarkers capture many aspects of muscle metabolism including mitochondria energy chain, protein synthesis, and oxidative stress. Our metabolomic analysis also revealed other important biomarkers such as L-arginine and aspartate. Although we recognize the importance of these metabolite biomarkers, both L-arginine and aspartate were difficult to detect using urinalysis.

L-glutamic acid and succinate play central roles in muscle metabolism by supporting ATP production and nitrogen balance, both which are critical for muscle function and adaptation (Zhang et al., 2023). Xanthine, a purine degradation product, was also identified as a metabolite of interest in both males and females. Beyond metabolic regulation, xanthine is related to oxidative stress and elevation of reactive oxygen species (ROS), processes which contribute to muscle fiber damage and impaired regeneration (Booth, 2000; Park et al., 2004; Sánchez-Sánchez et al., 2024). Xanthine is increased in oxidative stress and metabolic strain, which may also contribute to sarcopenic progression by impairing mitochondrial function and accelerating protein degradation (Park et al., 2004; Sánchez-Sánchez et al., 2024). Taurine, another key metabolite in our panel, plays a crucial role in muscle energy metabolism, while its anti-inflammatory and antioxidant properties support muscle health across different activity levels and disease states (Wen et al., 2019; Warskulat et al., 2004; De Luca et al., 2015). Unlike previous approaches that relied on single metabolites (De Luca et al., 2015), our study highlights the value of a multi-biomarker panel for predicting sarcopenia. Furthermore, urinary biomarkers provide an accessible and non-invasive tool for assessing muscle mass and function making them more feasible for routine clinical use. We detected several carnitines bound to fatty acids in our metabolomic analysis. This metabolite is usually found inside mitochondria promoting fatty acid transport for energy generation very important for muscle function (McCann et al., 2021; Longo et al., 2006).

While most individual metabolites in our panel did not exhibit statistically significant differences between sarcopenic and non-sarcopenic groups, the combined biomarker panel showed a much stronger discriminatory performance, as reflected by the high AUC values in our ROC analysis. The five metabolites were selected based on their established roles in interconnected metabolic pathways governing muscle energy metabolism, mitochondrial function, oxidative stress, and protein turnover, and therefore should not be considered independent predictors. Accordingly, the model was designed to evaluate the discriminatory capacity of a coordinated metabolic signature rather than to establish a diagnostic classifier, as multivariate signatures can exhibit discriminatory performance despite limited univariate differences in biologically coupled systems (Wang et al., 2024). This observation is consistent with prior metabolomics studies showing that panels of multiple biomarkers outperform single-metabolite approaches in complex, multifactorial conditions (Lee et al., 2023). Notably, the lack of statistical significance at the individual metabolite level, particularly after correction for multiple comparisons, does not preclude informative discriminatory value when biologically interdependent metabolites are evaluated jointly. As demonstrated by others (Montero et al., 2023; Zhan et al., 2017), analytical effect size does not necessarily correspond to biological impact in metabolically coupled systems. In metabolic and signaling networks, modest perturbations in upstream or intermediary metabolites can propagate through enzymatic cascades and regulatory feedback loops, such that biologically meaningful differences may only emerge when metabolites are considered collectively rather than in isolation. The underlying reason for this is that sarcopenia is a complex, multifactorial condition involving diverse metabolic pathways, including mitochondrial function, amino acid metabolism, oxidative stress, and protein turnover (Meng and Yu, 2010). When these aspects are analyzed together, metabolites collectively reflect shifts in metabolic pathways, capturing subtle metabolic changes that may not be evident from single metabolite analysis. This principle is well established in metabolic network analyses, where metabolites often exhibit nonlinear relationships and interact through compensatory mechanisms (Papp et al., 2009). This approach enhances the predictive power of a panel of biomarkers for identifying early sarcopenia, offering potential targets for future therapeutic interventions aimed at sustaining muscle health.

Importantly, using our biomarker panel to assess sarcopenia we achieved similar AUC comparing to DXA alone and physical assessments, this finding shows that our panel not only detect sarcopenia similar to golden standards but also reflects data on overall muscle mass and physical function. Therefore, our panel of biomarkers could provide a more consistent and reliable indicator of muscle health via urinalysis, reducing the need for more invasive and resource-intensive procedures. Additionally, we analyzed the biomarkers using volcanos plots. Although effect sizes do not always align with biological relevance, these plots still provided useful insights. Indeed, even signals with relatively small effect sizes may play pivotal roles by triggering downstream cascades within key metabolic pathways (Ebrahimpoor and Goeman, 2021). In our analysis, L-glutamic acid and xanthine showed the highest effect sizes among males, while xanthine and oleoylcarnitine were most prominent in females.

It is important to further emphasize the utility of urinalysis for muscle health monitoring. Urinalysis has emerged as an accessible diagnostic tool for a variety of conditions, ranging from the early detection of pancreatic cancer to the assessment of renal health (Debernardi et al., 2023; Libório et al., 2014; Oterdoom et al., 2008). Particularly in muscular diseases such as myotonic dystrophy and Duchenne muscular dystrophy, extracellular RNA markers in urine offer a non-invasive monitoring approach (Antoury et al., 2018). Furthermore, in rheumatoid arthritis, urinary metabolites such as oxoisovalerate, dimethylglycine, and isobutyric acid have been identified as indicators of reduced skeletal muscle mass (Oliveira et al., 2023). Urinalysis can also be used to monitor muscle health in healthy individuals and can be incorporated into routine clinical practice for disease prevention. For instance, proteins such as CTSH, PIK3IP1, DEFB1, ITGB1, BCAN, and TNFRSF10C when present in urine have proven useful in detecting muscle damage in healthy individuals, as seen in cases of exertional rhabdomyolysis (Carneiro et al., 2022). Biomarkers such as titin N-fragment and creatinine have provided insights into muscle degradation, reflecting the metabolic state of muscle tissue (Heymsfield et al., 1983; Baxmann et al., 2008; Oterdoom et al., 2008). However, the reliability of these proteins in urine samples remains limited for clinical use and understanding of muscle condition, as their levels can be affected by factors other than muscle health, particularly in populations with high variability in muscle mass, such as the elderly. In addition, the urine levels of these proteins are highly dependent on renal function (Heymsfield et al., 1983; Sheedy et al., 2014; Picca et al., 2021). As metabolite panels become validated in larger cohorts, advancements in assay development and automation could reduce costs, enabling integration into standard laboratory workflows. Urine-based biomarker testing, due to its non-invasive nature, can be bundled with routine primary care checkups, making it a practical tool for early sarcopenia detection and muscle health monitoring.

**FIGURE 9:**
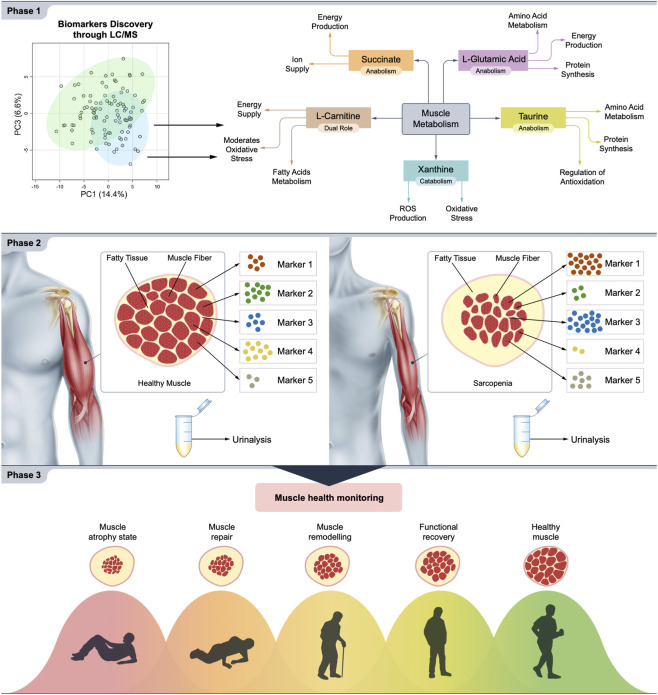
Summary of biomarker discovery and application in monitoring muscle health. Phase 1: Biomarkers linked to muscle health (taurine, xanthine, L-glutamic acid, succinate, and L-carnitine) were identified from a human tissue bank using LC/MS. Phase 2: On the left, a healthy individual with a biomarker profile suggestive of normal muscle function, and on the right, an individual with sarcopenia, highlighting biomarkers that can be analyzed to assess sarcopenia. This panel emphasizes the predictive potential of the biomarker panel for sarcopenia validated against the gold-standard DXA assessment for muscle mass. Phase 3: This phase showcases the prognostic utility of the biomarker panel by mapping biomarker levels from the onset of muscle injury through recovery.

Overall, this study underscores the importance of considering the multifaceted aspects of muscle health and their interplay in clinical practice. Understanding the relationships between muscle function, muscle mass, and muscle metabolism has implications for the prevention, diagnosis, and management of musculoskeletal disorders and age-related muscle decline. Through this approach, we successfully captured the complexity of sarcopenia, a multifactorial condition often challenging to assess accurately. In Figure 9, we summarize our research approach and future directions, using generic labels like “Marker 1” and “Marker 2” to emphasize that no single biomarker can reliably predict sarcopenia. Instead, our findings highlight the strength of analyzing biomarkers as a panel, which collectively provides greater sensitivity and specificity than individual markers, reinforcing the utility of this panel as a comprehensive predictive tool for sarcopenia.

We acknowledge limitations in this study, given the limited number of sarcopenic cases, and although, leave-one-out cross-validation demonstrated stable discrimination, this internal validation does not eliminate small-sample optimism, and independent external validation in larger sarcopenic cohorts is required prior to clinical translation. Accordingly, the present analysis is a proof-of-concept of muscle-related metabolic signatures rather than a definitive assessment of sarcopenia diagnosis. In addition, medication use and short-term metabolic variability were not controlled in this study, although urine dilution was assessed using urine density measurements and found to be consistent across participants. Future studies will investigate how comorbid conditions, such as cardiovascular and musculoskeletal diseases influence biomarker levels, helping to elucidate how these diseases may confound or modify biomarker-based detection of sarcopenia. Future research should incorporate longitudinal analyses and interventional trials, such as resistance training or nutritional modifications, to assess how biomarker levels respond to targeted treatments. By building on this foundational research will enable urinary metabolic signatures as a scalable, non-invasive solution for monitoring muscle health and identifying early metabolic changes associated with sarcopenia in aging populations.

## Data Availability

The raw data supporting the conclusions of this article will be made available by the authors, without undue reservation.
