# Supplementary material for: Metabolite biomarkers present in urine predict alterations in skeletal muscle associated with sarcopenia
Source: Front Aging. 2026 Mar 20;7:1736916. doi: 10.3389/fragi.2026.1736916 (PMC13047149; doi:10.3389/fragi.2026.1736916)
Supplement: Supplementary file 1 [file DataSheet2.pdf]

Supplemental Table 1. Leave-one-out cross-validation (LOO-CV) performance metrics for the multivariable logistic regression model.

AUC	Std Error	95% Confidence interval	P Value
0.8872	0.04923	0.7907 to 0.9837	<0.0001
0.8916	0.04903	0.7955 to 0.9877	<0.0001
0.8846	0.05028	0.7861 to 0.9832	<0.0001
0.8864	0.05006	0.7883 to 0.9845	<0.0001
0.8846	0.05028	0.7861 to 0.9832	<0.0001
0.8899	0.04885	0.7941 to 0.9856	<0.0001
0.8963	0.04740	0.8034 to 0.9892	<0.0001
0.8881	0.04909	0.7919 to 0.9843	<0.0001
0.8916	0.04904	0.7955 to 0.9877	<0.0001
0.9038	0.04546	0.8147 to 0.9929	<0.0001
0.8881	0.04996	0.7902 to 0.9860	<0.0001
0.8951	0.04612	0.8047 to 0.9855	<0.0001
0.8846	0.05028	0.7861 to 0.9832	<0.0001
0.9073	0.04437	0.8204 to 0.9943	<0.0001
0.8916	0.04707	0.7993 to 0.9839	<0.0001
0.9222	0.04025	0.8433 to 1.000	<0.0001
0.8864	0.05006	0.7883 to 0.9845	<0.0001
0.9021	0.04809	0.8078 to 0.9964	<0.0001
0.8833	0.05300	0.7795 to 0.9872	<0.0001
0.8846	0.05028	0.7861 to 0.9832	<0.0001
0.8846	0.05028	0.7861 to 0.9832	<0.0001
0.9148	0.04813	0.8205 to 1.000	<0.0001
0.8846	0.05028	0.7861 to 0.9832	<0.0001
0.8981	0.04713	0.8058 to 0.9905	<0.0001
0.9091	0.04436	0.8222 to 0.9960	<0.0001
0.8934	0.04884	0.7976 to 0.9891	<0.0001
0.8846	0.05028	0.7861 to 0.9832	<0.0001
0.8969	0.04496	0.8087 to 0.9850	<0.0001
0.9126	0.04267	0.8290 to 0.9962	<0.0001
0.8864	0.05005	0.7883 to 0.9845	<0.0001
0.9074	0.04047	0.8281 to 0.9867	<0.0001
0.8963	0.05139	0.7956 to 0.9970	<0.0001
0.8889	0.04968	0.7915 to 0.9863	<0.0001
0.8872	0.04923	0.7907 to 0.9837	<0.0001
0.8778	0.04944	0.7809 to 0.9747	<0.0001
0.8926	0.05049	0.7936 to 0.9915	<0.0001
0.8846	0.05028	0.7861 to 0.9832	<0.0001
0.9038	0.04580	0.8141 to 0.9936	<0.0001
0.8881	0.04988	0.7903 to 0.9859	<0.0001
0.8846	0.05028	0.7861 to 0.9832	<0.0001
0.8846	0.05028	0.7861 to 0.9832	<0.0001
0.8872	0.04923	0.7907 to 0.9837	<0.0001
0.8986	0.04430	0.8118 to 0.9854	<0.0001
0.8899	0.04804	0.7957 to 0.9840	<0.0001
0.8846	0.05028	0.7861 to 0.9832	<0.0001
0.8796	0.05226	0.7772 to 0.9821	<0.0001
0.8934	0.04939	0.7965 to 0.9902	<0.0001
0.8881	0.04833	0.7934 to 0.9828	<0.0001
0.8846	0.05028	0.7861 to 0.9832	<0.0001
0.8846	0.05028	0.7861 to 0.9832	<0.0001
0.8916	0.04816	0.7972 to 0.9860	<0.0001
0.8916	0.04741	0.7987 to 0.9845	<0.0001
0.8759	0.05415	0.7698 to 0.9821	<0.0001
0.9108	0.04381	0.8250 to 0.9967	<0.0001
0.8864	0.05006	0.7883 to 0.9845	<0.0001
0.8846	0.05028	0.7861 to 0.9832	<0.0001
0.8951	0.04615	0.8046 to 0.9856	<0.0001
0.8951	0.04834	0.8004 to 0.9898	<0.0001
0.8846	0.05028	0.7861 to 0.9832	<0.0001
