# Supplementary material for: Metabolite biomarkers present in urine predict alterations in skeletal muscle associated with sarcopenia
Source: Front Aging. 2026 Mar 20;7:1736916. doi: 10.3389/fragi.2026.1736916 (PMC13047149; doi:10.3389/fragi.2026.1736916)
Supplement: Supplementary file 2 [file DataSheet1.pdf]

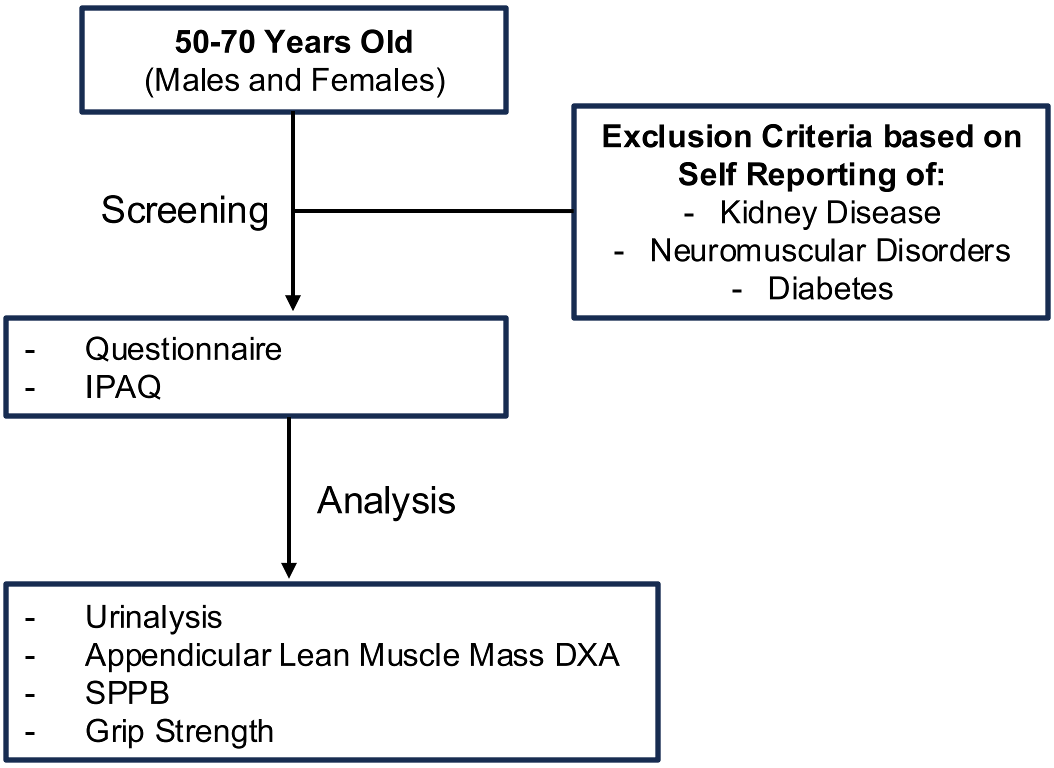

Supplemental Fig. 1. Prospective Clinical Evaluation Study. This investigation encompassed a cross-sectional study, wherein a comprehensive analysis of muscle function, muscle mass, and muscle metabolism was undertaken. The objective was to delineate the intricate relationships between these factors and elucidate their reciprocal influences on our understanding of muscle health. We identified and correlated metabolites with quantity and quality of muscle to predict sarcopenia. IPAQ: International physical assessment questionnaire, SPPB: Standard physical performance battery, DXA: dual X-ray absorptiometry. Samples were collected and immediately frozen to preserve metabolites. All samples were obtained before the physical assessment tests after 8h of fasting, with the first urination event of the morning collected. Participants were allowed to have breakfast before completing the physical assessments.
